# Varicose Veins and Their Complications.—III

**Published:** 1897-11-06

**Authors:** W. McAdam Eccles

**Affiliations:** Assistant-Surgeon West London Hospital, City of London Truss Society, &c.


### MEDICAL PROGRESS AND HOSPITAL CLINICS.

[*The Editor will be glad to receive offers of co-operation and contributions from members of the profession. All letters should be addressed to* THE EDITOR, AT THE OFFICE, 28 & 29, SOUTHAMPTON STREET, STRAND, LONDON, W.C.]

### VARICOSE VEINS AND THEIR COMPLICATIONS.—III.

(*Continued from page* 43.)

By W. McADAM ECCLES, M.S. (Lond.), F.R.C.S. (Eng.), Assistant-Surgeon West London Hospital, City of London Truss Society, &c.

The subject of ulceration dependent upon varicosity would be incomplete without reference to the by no means infrequent and serious *hæmorrhage* which may result from the ulcerative process extending into the dilated vein itself. In the greater number of cases owing to phlebitis, thrombosis of the affected vein occurs, and thus bleeding is not likely to ensue, the vessel being securely plugged; but in some cases this plugging does not act efficiently, and blood issues forth. The amount thus lost may be very great, and that in a comparatively short space of time; in fact, fatal syncope may be induced.

The cause of this excessive hæmorrhage is not difficult to determine. The vein has its calibre much increased, and, owing to the fibroid change in its walls, it has but little tendency to collapse when opened into. Moreover, since the valves are incompetent, the patient is in reality losing blood from the proximal portion of the venous channel, and might almost be said to be bleeding from the femoral vein. The treatment of such cases is simple and very efficacious, but needs to be promptly applied. The patient should be directed to lie down in a horizontal position, and the surgeon or any bystander must at once elevate the limb to a right angle with the trunk. This will immediately bring about a cessation of the blood flow, for seeing that blood has been pouring out owing to the dependent position, elevation must of necessity stop the stream. A tight band, applied round the limb on the proximal side of the ulcer, would also be effective in the case of a varicose vein, although most mischievous in other forms of venous hæmorrhage.

After elevation as a temporary measure, thorough cleansing and antiseptic dressing of the ulcer and surrounding area, together with moderate pressure, should be followed out. By this means healing of the ulcer, and consequently of the vein, will be brought about.

In some rare cases a chronic varicose ulcer may become the site of a malignant growth, epithelioma more commonly, but occasionally a sarcoma. It is seldom that amputation is required for chronic ulcers, except when they have led to the production of a kind of spurious elephantiasis of the foot, or have themselves taken on malignant characters.

Varix of the internal saphenous vein high up in the thigh, shortly before it passes through the saphenous opening, is liable to be mistaken for a femoral hernia. There are, however, certain points which should aid in their differential diagnosis. The vein has a much softer and more indefinite feel on palpation, the impulse on cough is rather of the nature of a fluid thrill, and when reduction is attempted in the upright position no solid body can be felt to slip away from the fingers. Moreover, if the patient be directed to lie down, the whole of the swelling will spontaneously disappear. If now the surgeon places his finger over the region of the femoral ring, and keeps it there while the patient rises to the erect posture, the swelling will reappear if it be a dilated vein, since this will fill from below, but a hernia would be prevented from descending. Also the fact of distal varicose veins points to the true nature of the case. The treatment of a vein thus enlarged in this region is not very satisfactory. The patient often complains of a dull aching, and this may in some cases be partially relieved by the application of an accurately fitting light femoral truss. But usually the patient has the veins dilated at a lower part of the limb, though it is not common to find that portion of the vein between the saphenous opening and the knee dilated. In such a case operative procedure, if the symptoms make it necessary, is indicated.

It has been necessary to review the ætiology and pathology of varicose veins in order to be able to treat them rationally. Mere dilatation due to plugging of deep veins needs no treatment beyond slight support, and, if occurring in the lower extremity, warning must be given of the serious harm which may be done by the wearing of a tight band around the lower part of the thigh. It goes without saying that to ligature or excise such dilated superficial veins is worse than useless, for it would be positively dangerous, and might even lead to gangrene.

Again, varicosity of the superficial veins in a well-developed muscular subject but rarely needs attention, and then most commonly only in the form of some elastic support. In fact, it may be taken as a useful general rule that, unless varicose veins give rise to symptoms or lead to complications, they seldom need active treatment. When, however, one is confronted with a patient of not too strong a physique, forced to undergo many hours in the upright position, and showing a marked varicosity of the veins of the lower extremity, accompanied by symptoms but no complications, it is then that treatment is indicated which will lead to the greatest benefit. Such treatment may be divided into the palliative and the operative.

The palliative form of treatment consists for the most part of uniform and constant pressure upon the superficial dilated veins whenever the patient assumes the standing or sitting position, pressure in the recumbent position not being needed. It is absolutely essential that, while this general pressure is being exercised, no local constriction of any kind be superadded, and that, whenever possible, the horizontal position should be given to the affected limb. While such local palliative measures are being employed, constitutional treatment in the form of tonics, such as iron, strychnine, &c., should be administered; a healthy, firm tone to the body generally, and the muscles in particular, will thereby be secured.

With regard to the best means of employing pressure to varicose veins it is difficult to lay down any general rule. Elastic stockings are very frequently prescribed, and, no doubt, in suitable cases they may relieve symptoms. They have, however, two serious objections, one being that they are likely to be too tight above and perhaps not tight enough below, and the other is that they are very likely to become useless in a comparatively short space of time on account of their great tendency to stretch. A pure rubber bandage is also used, but although acting probably much more efficiently than an elastic stocking, yet they are very liable to produce eczema or irritation of the skin, because they confine all the excretion of the skin. The perforated variety of this bandage, which is supposed to obviate this retention, is actually more harmful, causing much greater irritation. I have found the application of a gauze bandage from the foot to the knee, or higher, to be one of the most comfortable and satisfactory methods of applying uniform pressure without retention of skin excretions. It requires to be rather frequently renewed however; but its cheapness and efficiency counterbalances, I think, this objection.

In a considerable number of cases, however, palliative measures either fail or are too irksome to the patient to be satisfactorily carried out. Operative treatment will then become advisable. Before proceeding to carry this out, several considerations have to be paid attention to. Firstly, the superficial veins must on no account be obliterated if there is any indication that the deep veins are plugged. This fact can usually be determined by careful attention to the history of the case; it has been alluded to in the first section of this paper. Secondly, the patient's general health, age, sex, and occupation should be taken into account. A debilitated condition will materially influence the success of any radical operation, but very especially so that for varicose veins. The younger the patient the more likely is the result to be a satisfactory one. A sedentary occupation will usually militate against a cure.

As to the operation to be undertaken, the method of exposing the vein and ligaturing it in two places and dividing between has certainly the most to recommend it. This procedure will have to be carried out at numerous spots if the length of vein which is varicosed be considerable.

Much attention must be paid to ensure asepsis before the operation is performed. The limb should be shaved, scrubbed with soap and water, washed over with turpentine, and then with a solution of 1·2,000 perchloride of mercury solution. Before the antiseptic dressing is put on the limb should be allowed to hang dependent (without touching any septic substance), or the patient directed to stand erect, to cause the veins to become prominent. An aniline pencil is then moistened, and the several spots where it is intended to ligature the vein touched so that a mark may be left on the skin. It is not necessary to mark out any length of the vein; in fact, this is confusing. The antiseptic dressing of 1-2,000 perchloride or biniodide of mercury is securely applied from the foot to above the highest spot at which the vein is to be obliterated. I prefer, if possible, that the whole of the above preparation should be repeated twenty-four hours after it was first carried out. It is certain in this way that the contents of the skin glands which will have come to the surface will be washed away, and the skin again freshly covered with an antiseptic dressing which remains on until the actual time of the operation.

While the patient is being anæsthetised it is a good plan to allow the affected limb to hang over the side of the operating table, in order that the veins may become distended. The dressing is removed, and the surface washed over again with antiseptic solution. The first point at which the vein should be ligatured should be the most proximal one, for in this way the veins below will be kept filled with blood, and there will be no likelihood of clot being carried into the circulation. An incision is made by the side of the vein, exposing about an inch of it; this is dissected cleanly from the surrounding tissue, and an aneurism needle passed under. A length of aseptic twist silk, sufficiently long for two ligatures, is then drawn behind the vein by threading and withdrawing the needle. The loop of this is cut, and the vein tied above and below, and then about half an inch of its length between the two ligatures is cut away with scissors. The small skin incision is accurately sutured with fine salmon gut, and an antiseptic dressing applied. A like operation is carried out at each spot where it has been considered necessary to obliterate the vein. Finally, firm pressure is maintained over the whole limb by the application of several layers of carbolic gauze bandage. The dressing should be left untouched until the seventh or eighth day, when it may be removed and the sutures cut out. Complete healing should have occurred. The patient may sit up on the tenth day, but should not be allowed to walk about until the twelfth.

The same result as that obtained by ligaturing the vein and suturing the skin may be brought about by applying forcipressure forceps to the vein at the points where a ligature would be applied, and leaving them on for some ten or fifteen minutes, and by merely drawing the edges of the skin wound together by isinglass plaster. This obviates the necessity of removing sutures, but the method is not quite so certain as that above described. After operation upon varicose veins a patient should wear a gauze bandage support for a few months, until the parts have been restored to more or less their normal condition. These operative measures are in the majority of instances followed by the most beneficial and satisfactory results.

